# Evaluation of automated contour detection to produce regional delay maps from high temporal resolution cine images in patients undergoing CRT

**DOI:** 10.1186/1532-429X-16-S1-P373

**Published:** 2014-01-16

**Authors:** Patrick Magrath, Jonathan D Suever, Rob J van der Geest, John Oshinski

**Affiliations:** 1 Radiology and Imaging Sciences, Emory University School of Medicine, Atlanta, Georgia, USA; 2 Wallace H. Coulter Department of Biomedical Engineering, Georgia Institute of Technology, Atlanta, Georgia, USA; 3 Department of Radiology, Leiden University Medical Center, Leiden, Netherlands

## Background

Recently, high temporal resolution short-axis cine images have been used to create left ventricular (LV) regional mechanical delay (RMD) maps in patients undergoing cardiac resynchronization therapy (CRT). Generating the endocardial segmentation required for RMD analysis is both time-consuming and subjective. A semi-automated segmentation method for time-continuous contour detection that uses multi-dimensional dynamic programming to enforce user-defined shape constraints without the need for extensive training sets has been previously developed (Üzümcü 2006). Our goal was to compare RMD maps generated from semi-automated and manual segmentation in patients enrolled for CRT.

## Methods

Manual LV endocardial segmentations were compared to contours generated by the semi-automated segmentation method using every 4th, 8th, or 12th contour (auto4, auto8, auto12, respectively) in 11 patients scheduled for CRT. Contour agreement was assessed using a signed Euclidean distance-based shape similarity metric (Pluempitiwiriyawaj 2005) where 100% represents perfect agreement. RMD maps generated from each segmentation method were compared by computing the average delay difference between maps and identification the latest contracting AHA segment.

## Results

Shape similarity between the reference contour set and auto-generated contour sets was high (90.4% - 87.8%), table 1. There were small non-significant differences in delay times between the semi-automated RMD maps (p = .8330, .4109, .3524), but there were significant differences between mechanical delays from semi-automated and manual segmentation (p < 0.01). The latest contracting AHA segment agreed in 88% of the auto8 and auto12 patients and in 66% of the auto4 group. For the remaining 22% and 34% of patient respectively, the latest contracting segment was in an adjacent segment.

**Table 1:** 

				Latest Segment Agreement with References	
	**Shape Similarity (%)**	**Delay Difference (%CC)**	**P-value Auto vs. Reference**	**Same**	**Same or Adjacent**

Auto 4	90 ± 6	7 ± 10	0.01	66% (8/11)	100%

Auto 8	89 ± 5	5 ± 10	< 0.01	88% (9/11)	100%

Auto 12	88 ± 4	10 ± 12	< 0.001	88% (9/11)	100%

Comparison of semi-automatic and manual segmentation using shape similarity, bullseye subtraction, and identification of region of latest contraction.

## Conclusions

We identified small differences between semi-automated and manual segmentation of high temporal resolution images of the LV. However, these differences do not significantly affect the identification of the region of latest contraction. Integration of semi-automated segmentation with RMD analysis warrants examination as a workflow enhancement in patients undergoing MRI before CRT.

## Funding

Grants from American Heart Association (AHA) and National Institutes of Health (NIH) to John Oshinski. Jonathan Suever was supported by a National Science Foundation (NSF) Fellowship.

**Figure 1:**
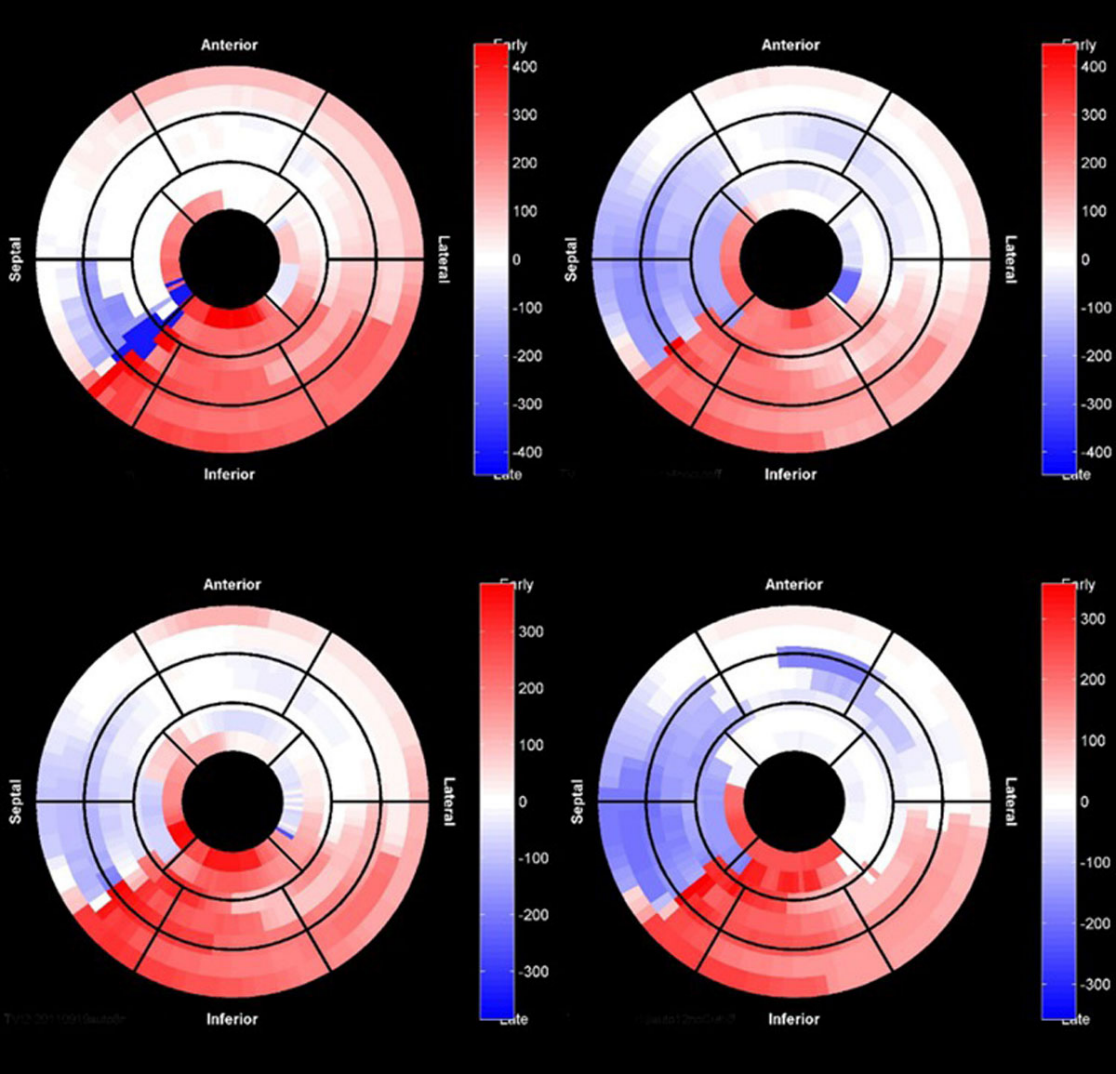
**Representative patient with RMD assessed using manual segmentation (top left) and semi-automated segmentation using every fourth (auto4, top right), eighth (auto8, bottom left), and twelfth (auto12, bottom right) contour**.

